# Supplementary material for: Extracting the femtometer structure of strange baryons using the vacuum polarization effect
Source: Nat Commun. 2024 Oct 11;15:8812. doi: 10.1038/s41467-024-51802-y (PMC11470094; doi:10.1038/s41467-024-51802-y)
Supplement: Supplementary file 1 — Supplementary Information [file 41467_2024_51802_MOESM1_ESM.pdf]

Extracting the femtometer structure of strange baryons using the vacuum polarization effect

The BESIII Collaboration*

(Dated: July 11, 2024)

Supplementary Information

Figures and Tables used in paper are listed accordingly.

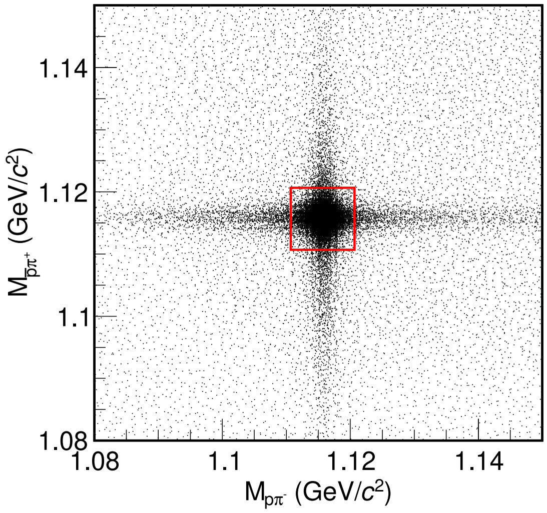

Supplementary Fig. 1 | Distribution of $M_{p\pi^-}$ versus $M_{\bar{p}\pi^+}$ of the accepted candidates from data. The red box denotes the signal region. The clusters of Λ and $\bar{\Lambda}$ are clearly observed.

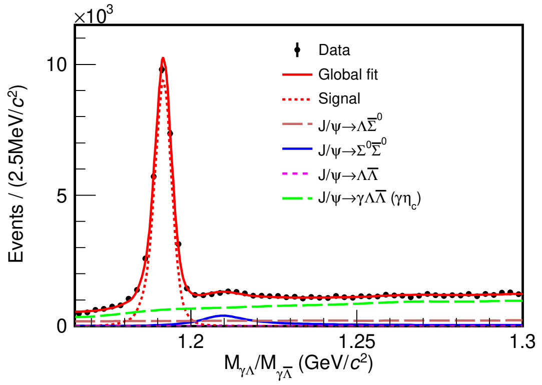

Supplementary Fig. 2 | Fit to the $M_{\gamma\Lambda}/M_{\gamma\bar{\Lambda}}$ distribution of the accepted candidates from J/ψ data. The black points with error bars are data, the red curve is the global fit, the red dotted curve is the $\Sigma^0/\bar{\Sigma}^0$ signal, the brown long-dashed curve shows the background from the conjugate channel, the blue curve is from $J/\psi \to \Sigma^0\bar{\Sigma}^0$, the pink dotted curve is from $J/\psi \to \Lambda\bar{\Lambda}$ and the green long-dashed curve is from $J/\psi \to \gamma\Lambda\bar{\Lambda}(\gamma\eta_c)$.

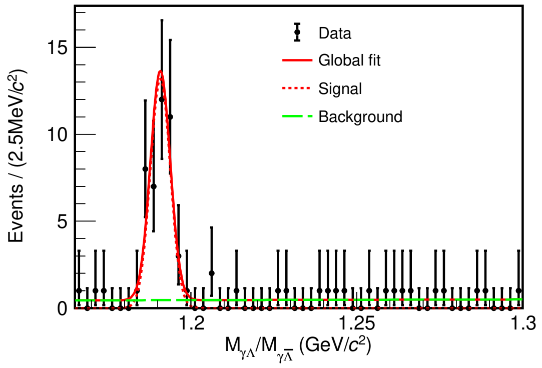

Supplementary Fig. 3 | Fit to the $M_{\gamma\Lambda}/M_{\gamma\bar{\Lambda}}$ distribution from the QED background. The black points with error bars are data, the red curve is the global fit, the red dotted curve is the signal shape described by a Breit-Wigner shape convolved with a Gaussian function and the green long-dashed curve is from the background described with a first-order Chebychev function.

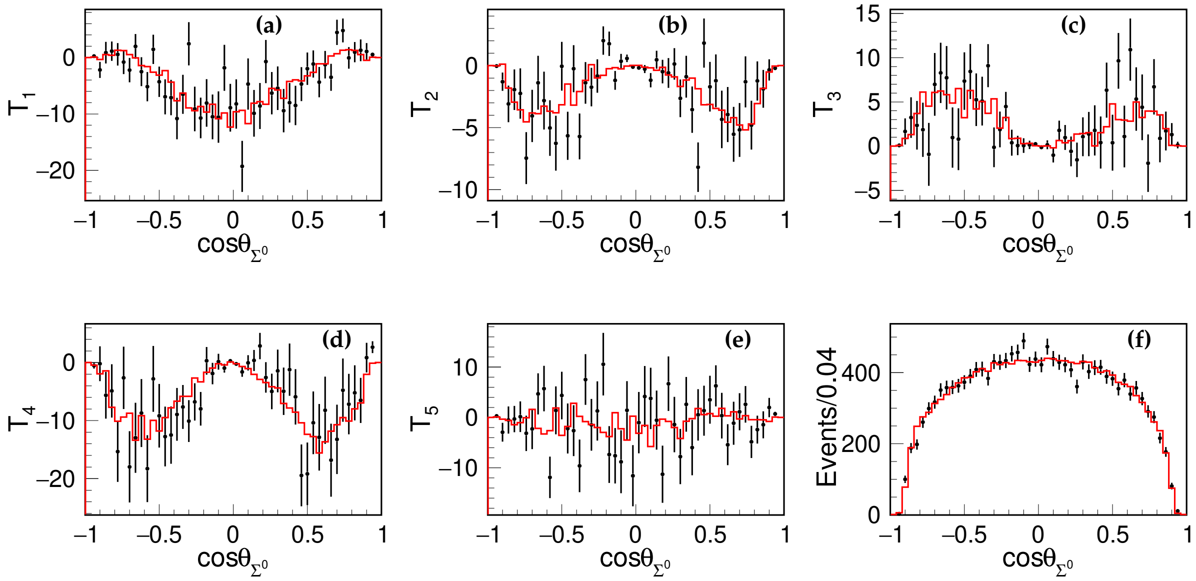

Supplementary Fig. 4 | Fit results compared with data of $e^+e^- \to J/\psi \to \bar{\Lambda}(\to \bar{p}\pi^+)\,\Sigma^0(\to \gamma\Lambda)$ process. (a)-(e) are the moments T_1-T_5 respectively and (f) is the Σ^0 angular distribution. The black points with error bars are data and the red curve is the global fit.

Supplementary Table 1 | Systematic uncertainties for the parameters measurement.

Source	$\alpha_{J/\psi}$	$\Delta\Phi_{\bar{\Lambda}\Sigma^0}$ (rad)	$\Delta\Phi_{\Lambda\bar{\Sigma}^0}$ (rad)
$\Lambda/\bar{\Lambda}$ reconstruction	0.0047	0.0038	0.0036
Kinematic fit	0.0010	0.0040	0.0049
Background estimate	0.0083	0.0065	0.0070
$\alpha_{\Lambda/\bar{\Lambda}}$	0.0000	0.0050	0.0019
Total	0.014	0.010	0.010

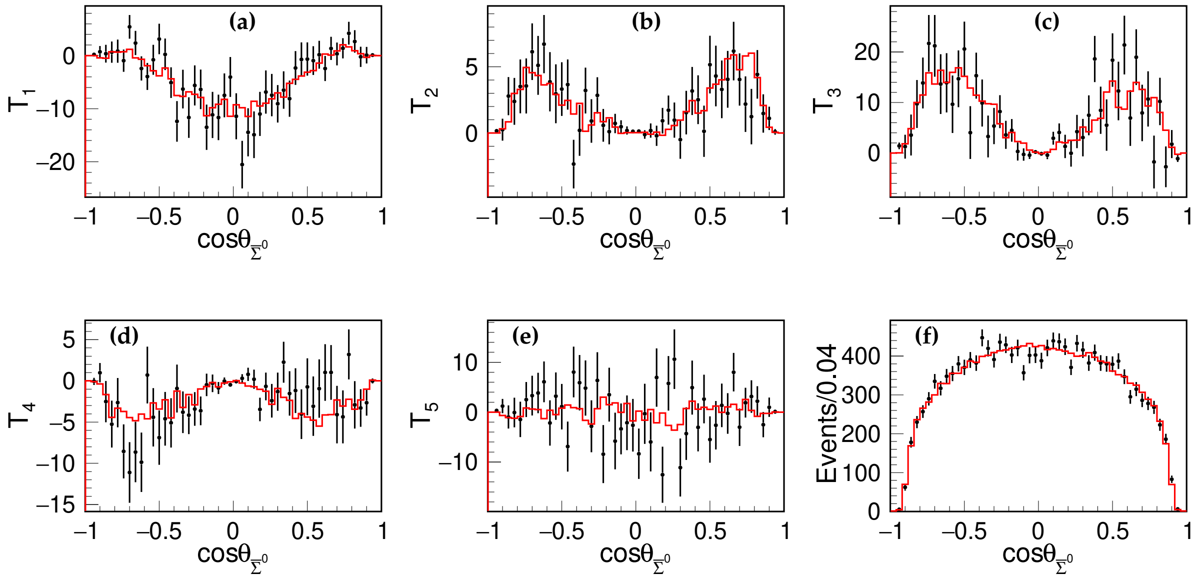

Supplementary Fig. 5 | Fit results compared with data of $e^+e^- \to J/\psi \to \Lambda(\to p\pi^-)\,\bar{\Sigma}^0(\to \gamma\bar{\Lambda})$ process. (a)-(e) is the moments T_1-T_5 respectively and (f) is the $\bar{\Sigma}^0$ angular distribution. The black points with error bars are data and the red curve is the global fit

The BESIII Collaboration

M. Ablikim[1], M. N. Achasov[12,b], P. Adlarson[72], M. Albrecht[4], R. Aliberti[33], A. Amoroso[71A,71C], M. R. An[37], Q. An[68,55], Y. Bai[54], O. Bakina[34], R. Baldini Ferroli[27A], I. Balossino[28A], Y. Ban[44,g], V. Batozskaya[1,42], D. Becker[33], K. Begzsuren[30], N. Berger[33], M. Bertani[27A], D. Bettoni[28A], F. Bianchi[71A,71C], E. Bianco[71A,71C], J. Bloms[65], A. Bortone[71A,71C], I. Boyko[34], R. A. Briere[5], A. Brueggemann[65], H. Cai[73], X. Cai[1,55], A. Calcaterra[27A], G. F. Cao[1,60], N. Cao[1,60], S. A. Cetin[59A], J. F. Chang[1,55], W. L. Chang[1,60], G. R. Che[41], G. Chelkov[34,a], C. Chen[41], Chao Chen[52], G. Chen[1], H. S. Chen[1,60], M. L. Chen[1,55,60], S. J. Chen[40], S. M. Chen[58], T. Chen[1,60], X. R. Chen[29,60], X. T. Chen[1,60], Y. B. Chen[1,55], Z. J. Chen[24,h], W. S. Cheng[71C], S. K. Choi [52], X. Chu[41], G. Cibinetto[28A], F. Cossio[71C], J. J. Cui[47], H. L. Dai[1,55], J. P. Dai[76], A. Dbeyssi[18], R. E. de Boer[4], D. Dedovich[34], Z. Y. Deng[1], A. Denig[33], I. Denysenko[34], M. Destefanis[71A,71C], F. De Mori[71A,71C], Y. Ding[32], Y. Ding[38], J. Dong[1,55], L. Y. Dong[1,60], M. Y. Dong[1,55,60], X. Dong[73], S. X. Du[78], Z. H. Duan[40], P. Egorov[34,a], Y. L. Fan[73], J. Fang[1,55], S. S. Fang[1,60], W. X. Fang[1], Y. Fang[1], R. Farinelli[28A], L. Fava[71B,71C], F. Feldbauer[4], G. Felici[27A], C. Q. Feng[68,55], J. H. Feng[56], K Fischer[66], M. Fritsch[4], C. Fritzsch[65], C. D. Fu[1], H. Gao[60], Y. N. Gao[44,g], Yang Gao[68,55], S. Garbolino[71C], I. Garzia[28A,28B], P. T. Ge[73], Z. W. Ge[40], C. Geng[56], E. M. Gersabeck[64], A Gilman[66], K. Goetzen[13], L. Gong[38], W. X. Gong[1,55], W. Gradl[33], M. Greco[71A,71C], L. M. Gu[40], M. H. Gu[1,55], Y. T. Gu[15], C. Y Guan[1,60], A. Q. Guo[29,60], L. B. Guo[39], R. P. Guo[46], Y. P. Guo[11,f], A. Guskov[34,a], W. Y. Han[37], X. Q. Hao[19], F. A. Harris[62], K. K. He[52], K. L. He[1,60], F. H. Heinsius[4], C. H. Heinz[33], Y. K. Heng[1,55,60], C. Herold[57], G. Y. Hou[1,60], Y. R. Hou[60], Z. L. Hou[1], H. M. Hu[1,60], J. F. Hu[53,i], T. Hu[1,55,60], Y. Hu[1], G. S. Huang[68,55], K. X. Huang[56], L. Q. Huang[29,60], X. T. Huang[47], Y. P. Huang[1], Z. Huang[44,g], T. Hussain[70], N Hüsken[26,33], W. Imoehl[26], M. Irshad[68,55], J. Jackson[26], S. Jaeger[4], S. Janchiv[30], E. Jang[52], J. H. Jeong[52], Q. Ji[1], Q. P. Ji[19], X. B. Ji[1,60], X. L. Ji[1,55], Y. Y. Ji[47], Z. K. Jia[68,55], P. C. Jiang[44,g], S. S. Jiang[37], X. S. Jiang[1,55,60], Y. Jiang[60], J. B. Jiao[47], Z. Jiao[22], S. Jin[40], Y. Jin[63], M. Q. Jing[1,60], T. Johansson[72], S. Kabana[31], N. Kalantar-Nayestanaki[61], X. L. Kang[9], X. S. Kang[38], R. Kappert[61], M. Kavatsyuk[61], B. C. Ke[78], I. K. Keshk[4], A. Khoukaz[65], R. Kiuchi[1], R. Kliemt[13], L. Koch[35], O. B. Kolcu[59A], B. Kopf[4], M. Kuemmel[4], M. Kuessner[4], A. Kupsc[42,72], W. Kühn[35], J. J. Lane[64], J. S. Lange[35], P. Larin[18], A. Lavania[25], L. Lavezzi[71A,71C], T. T. Lei[68,k], Z. H. Lei[68,55], H. Leithoff[33], M. Lellmann[33], T. Lenz[33], C. Li[45], C. Li[41], C. H. Li[37], Cheng Li[68,55], D. M. Li[78], F. Li[1,55], G. Li[1], H. Li[49], H. Li[68,55], H. B. Li[1,60], H. J. Li[19], H. N. Li[53,i], J. Q. Li[4], J. S. Li[56], J. W. Li[47], Ke Li[1], L. J Li[1,60], L. K. Li[1], Lei Li[3], M. H. Li[41], P. R. Li[36,j,k], S. X. Li[11], S. Y. Li[58], T. Li[47], W. D. Li[1,60], W. G. Li[1], X. H. Li[68,55], X. L. Li[47], Xiaoyu Li[1,60], Y. G. Li[44,g], Z. X. Li[15], Z. Y. Li[56], C. Liang[40], H. Liang[1,60], H. Liang[68,55], H. Liang[32], Y. F. Liang[51], Y. T. Liang[29,60], G. R. Liao[14], L. Z. Liao[47], J. Libby[25], A. Limphirat[57], C. X. Lin[56], D. X. Lin[29,60], T. Lin[1], B. J. Liu[1], C. Liu[32], C. X. Liu[1], D. Liu[18,68], F. H. Liu[50], Fang Liu[1], Feng Liu[6], G. M. Liu[53,i], H. Liu[36,j,k], H. B. Liu[15], H. M. Liu[1,60], Huanhuan Liu[1], Huihui Liu[20], J. B. Liu[68,55], J. L. Liu[69], J. Y. Liu[1,60], K. Liu[1], K. Y. Liu[38], Ke Liu[21], L. Liu[68,55], Lu Liu[41], M. H. Liu[11,f], P. L. Liu[1], Q. Liu[60], S. B. Liu[68,55], T. Liu[11,f], W. K. Liu[41], W. M. Liu[68,55], X. Liu[36,j,k], Y. Liu[36,j,k], Y. B. Liu[41], Z. A. Liu[1,55,60], Z. Q. Liu[47], X. C. Lou[1,55,60], F. X. Lu[56], H. J. Lu[22], J. G. Lu[1,55], X. L. Lu[1], Y. Lu[7], Y. P. Lu[1,55], Z. H. Lu[1,60], C. L. Luo[39], M. X. Luo[77], T. Luo[11,f], X. L. Luo[1,55], X. R. Lyu[60], Y. F. Lyu[41], F. C. Ma[38], H. L. Ma[1], L. L. Ma[47], M. M. Ma[1,60], Q. M. Ma[1], R. Q. Ma[1,60], R. T. Ma[60], X. Y. Ma[1,55], Y. Ma[44,g], F. E. Maas[18], M. Maggiora[71A,71C], S. Maldaner[4], S. Malde[66], Q. A. Malik[70], A. Mangoni[27B], Y. J. Mao[44,g], Z. P. Mao[1], S. Marcello[71A,71C], Z. X. Meng[63], J. G. Messchendorp[13,61], G. Mezzadri[28A], H. Miao[1,60], T. J. Min[40], R. E. Mitchell[26], X. H. Mo[1,55,60], N. Yu. Muchnoi[12,b],

Y. Nefedov[34], F. Nerling[18,d], I. B. Nikolaev[12,b], Z. Ning[1,55], S. Nisar[10,l], Y. Niu [47], S. L. Olsen[60], Q. Ouyang[1,55,60], S. Pacetti[27B,27C], X. Pan[52], Y. Pan[54], A. Pathak[32], Y. P. Pei[68,55], M. Pelizaeus[4], H. P. Peng[68,55], K. Peters[13,d], J. L. Ping[39], R. G. Ping[1,60], S. Plura[33], S. Pogodin[34], V. Prasad[68,55], F. Z. Qi[1], H. Qi[68,55], H. R. Qi[58], M. Qi[40], T. Y. Qi[11,f], S. Qian[1,55], W. B. Qian[60], Z. Qian[56], C. F. Qiao[60], J. J. Qin[69], L. Q. Qin[14], X. P. Qin[11,f], X. S. Qin[47], Z. H. Qin[1,55], J. F. Qiu[1], S. Q. Qu[58], K. H. Rashid[70], C. F. Redmer[33], K. J. Ren[37], A. Rivetti[71C], V. Rodin[61], M. Rolo[71C], G. Rong[1,60], Ch. Rosner[18], S. N. Ruan[41], A. Sarantsev[34,c], Y. Schelhaas[33], C. Schnier[4], K. Schoenning[72], M. Scodeggio[28A,28B], K. Y. Shan[11,f], W. Shan[23], X. Y. Shan[68,55], J. F. Shangguan[52], L. G. Shao[1,60], M. Shao[68,55], C. P. Shen[11,f], H. F. Shen[1,60], W. H. Shen[60], X. Y. Shen[1,60], B. A. Shi[60], H. C. Shi[68,55], J. Y. Shi[1], Q. Q. Shi[52], R. S. Shi[1,60], X. Shi[1,55], J. J. Song[19], W. M. Song[32,1], Y. X. Song[44,g], S. Sosio[71A,71C], S. Spataro[71A,71C], F. Stieler[33], P. P. Su[52], Y. J. Su[60], G. X. Sun[1], H. Sun[60], H. K. Sun[1], J. F. Sun[19], L. Sun[73], S. S. Sun[1,60], T. Sun[1,60], W. Y. Sun[32], Y. J. Sun[68,55], Y. Z. Sun[1], Z. T. Sun[47], Y. X. Tan[68,55], C. J. Tang[51], G. Y. Tang[1], J. Tang[56], L. Y Tao[69], Q. T. Tao[24,h], M. Tat[66], J. X. Teng[68,55], V. Thoren[72], W. H. Tian[49], Y. Tian[29,60], I. Uman[59B], B. Wang[1], B. Wang[68,55], B. L. Wang[60], C. W. Wang[40], D. Y. Wang[44,g], F. Wang[69], H. J. Wang[36,j,k], H. P. Wang[1,60], K. Wang[1,55], L. L. Wang[1], M. Wang[47], M. Z. Wang[44,g], Meng Wang[1,60], S. Wang[14], S. Wang[11,f], T. Wang[11,f], T. J. Wang[41], W. Wang[56], W. H. Wang[73], W. P. Wang[68,55], X. Wang[44,g], X. F. Wang[36,j,k], X. L. Wang[11,f], Y. Wang[58], Y. D. Wang[43], Y. F. Wang[1,55,60], Y. H. Wang[45], Y. Q. Wang[1], Yaqian Wang[17,1], Z. Wang[1,55], Z. Y. Wang[1,60], Ziyi Wang[60], D. H. Wei[14], F. Weidner[65], S. P. Wen[1], D. J. White[64], U. Wiedner[4], G. Wilkinson[66], M. Wolke[72], L. Wollenberg[4], J. F. Wu[1,60], L. H. Wu[1], L. J. Wu[1,60], X. Wu[11,f], X. H. Wu[32], Y. Wu[68], Y. J Wu[29], Z. Wu[1,55], L. Xia[68,55], T. Xiang[44,g], D. Xiao[36,j,k], G. Y. Xiao[40], H. Xiao[11,f], S. Y. Xiao[1], Y. L. Xiao[11,f], Z. J. Xiao[39], C. Xie[40], X. H. Xie[44,g], Y. Xie[47], Y. G. Xie[1,55], Y. H. Xie[6], Z. P. Xie[68,55], T. Y. Xing[1,60], C. F. Xu[1,60], C. J. Xu[56], G. F. Xu[1], H. Y. Xu[63], Q. J. Xu[16], X. P. Xu[52], Y. C. Xu[75], Z. P. Xu[40], F. Yan[11,f], L. Yan[11,f], W. B. Yan[68,55], W. C. Yan[78], H. J. Yang[48,e], H. L. Yang[32], H. X. Yang[1], S. L. Yang[1,60], Tao Yang[1], Y. F. Yang[41], Y. X. Yang[1,60], Yifan Yang[1,60], M. Ye[1,55], M. H. Ye[8], J. H. Yin[1], Z. Y. You[56], B. X. Yu[1,55,60], C. X. Yu[41], G. Yu[1,60], T. Yu[69], X. D. Yu[44,g], C. Z. Yuan[1,60], L. Yuan[2], S. C. Yuan[1], X. Q. Yuan[1], Y. Yuan[1,60], Z. Y. Yuan[56], C. X. Yue[37], A. A. Zafar[70], F. R. Zeng[47], X. Zeng[6], Y. Zeng[24,h], X. Y. Zhai[32], Y. H. Zhan[56], A. Q. Zhang[1,60], B. L. Zhang[1,60], B. X. Zhang[1], D. H. Zhang[41], G. Y. Zhang[19], H. Zhang[68], H. H. Zhang[32], H. H. Zhang[56], H. Q. Zhang[1,55,60], H. Y. Zhang[1,55], J. L. Zhang[74], J. Q. Zhang[39], J. W. Zhang[1,55,60], J. X. Zhang[36,j,k], J. Y. Zhang[1], J. Z. Zhang[1,60], Jianyu Zhang[1,60], Jiawei Zhang[1,60], L. M. Zhang[58], L. Q. Zhang[56], Lei Zhang[40], P. Zhang[1], Q. Y. Zhang[37,78], Shuihan Zhang[1,60], Shulei Zhang[24,h], X. D. Zhang[43], X. M. Zhang[1], X. Y. Zhang[47], X. Y. Zhang[52], Y. Zhang[66], Y. T. Zhang[78], Y. H. Zhang[1,55], Yan Zhang[68,55], Yao Zhang[1], Z. H. Zhang[1], Z. L. Zhang[32], Z. Y. Zhang[41], Z. Y. Zhang[73], G. Zhao[1], J. Zhao[37], J. Y. Zhao[1,60], J. Z. Zhao[1,55], Lei Zhao[68,55], Ling Zhao[1], M. G. Zhao[41], S. J. Zhao[78], Y. B. Zhao[1,55], Y. X. Zhao[29,60], Z. G. Zhao[68,55], A. Zhemchugov[34,a], B. Zheng[69], J. P. Zheng[1,55], W. J. Zheng[1,60], Y. H. Zheng[60], B. Zhong[39], C. Zhong[69], X. Zhong[56], H. Zhou[47], L. P. Zhou[1,60], X. Zhou[73], X. K. Zhou[60], X. R. Zhou[68,55], X. Y. Zhou[37], Y. Z. Zhou[11,f], J. Zhu[41], K. Zhu[1], K. J. Zhu[1,55,60], L. X. Zhu[60], S. H. Zhu[67], S. Q. Zhu[40], T. J. Zhu[74], W. J. Zhu[11,f], Y. C. Zhu[68,55], Z. A. Zhu[1,60], J. H. Zou[1], J. Zu[68,55]

[1] *Institute of High Energy Physics, Beijing 100049, People's Republic of China.* [2] *Beihang University, Beijing 100191, People's Republic of China.* [3] *Beijing Institute of Petrochemical Technology, Beijing 102617, People's Republic of China.* [4] *Bochum Ruhr-University, D-44780 Bochum, Germany.* [5] *Carnegie Mellon University, Pittsburgh, Pennsylvania 15213, USA.* [6] *Central China*

Normal University, Wuhan 430079, People's Republic of China. [7] Central South University, Changsha 410083, People's Republic of China. [8] China Center of Advanced Science and Technology, Beijing 100190, People's Republic of China. [9] China University of Geosciences, Wuhan 430074, People's Republic of China. [10] COMSATS University Islamabad, Lahore Campus, Defence Road, Off Raiwind Road, 54000 Lahore, Pakistan. [11] Fudan University, Shanghai 200433, People's Republic of China. [12] G.I. Budker Institute of Nuclear Physics SB RAS (BINP), Novosibirsk 630090, Russia. [13] GSI Helmholtzcentre for Heavy Ion Research GmbH, D-64291 Darmstadt, Germany. [14] Guangxi Normal University, Guilin 541004, People's Republic of China. [15] Guangxi University, Nanning 530004, People's Republic of China. [16] Hangzhou Normal University, Hangzhou 310036, People's Republic of China. [17] Hebei University, Baoding 071002, People's Republic of China. [18] Helmholtz Institute Mainz, Staudinger Weg 18, D-55099 Mainz, Germany. [19] Henan Normal University, Xinxiang 453007, People's Republic of China. [20] Henan University of Science and Technology, Luoyang 471003, People's Republic of China. [21] Henan University of Technology, Zhengzhou 450001, People's Republic of China. [22] Huangshan College, Huangshan 245000, People's Republic of China. [23] Hunan Normal University, Changsha 410081, People's Republic of China. [24] Hunan University, Changsha 410082, People's Republic of China. [25] Indian Institute of Technology Madras, Chennai 600036, India. [26] Indiana University, Bloomington, Indiana 47405, USA. [27] INFN Laboratori Nazionali di Frascati , (A)INFN Laboratori Nazionali di Frascati, I-00044, Frascati, Italy; (B)INFN Sezione di Perugia, I-06100, Perugia, Italy; (C)University of Perugia, I-06100, Perugia, Italy. [28] INFN Sezione di Ferrara, (A)INFN Sezione di Ferrara, I-44122, Ferrara, Italy; (B)University of Ferrara, I-44122, Ferrara, Italy. [29] Institute of Modern Physics, Lanzhou 730000, People's Republic of China. [30] Institute of Physics and Technology, Peace Avenue 54B, Ulaanbaatar 13330, Mongolia. [31] Instituto de Alta Investigación, Universidad de Tarapacá, Casilla 7D, Arica, Chile. [32] Jilin University, Changchun 130012, People's Republic of China. [33] Johannes Gutenberg University of Mainz, Johann-Joachim-Becher-Weg 45, D-55099 Mainz, Germany. [34] Joint Institute for Nuclear Research, 141980 Dubna, Moscow region, Russia. [35] Justus-Liebig-Universitaet Giessen, II. Physikalisches Institut, Heinrich-Buff-Ring 16, D-35392 Giessen, Germany. [36] Lanzhou University, Lanzhou 730000, People's Republic of China. [37] Liaoning Normal University, Dalian 116029, People's Republic of China. [38] Liaoning University, Shenyang 110036, People's Republic of China. [39] Nanjing Normal University, Nanjing 210023, People's Republic of China. [40] Nanjing University, Nanjing 210093, People's Republic of China. [41] Nankai University, Tianjin 300071, People's Republic of China. [42] National Centre for Nuclear Research, Warsaw 02-093, Poland. [43] North China Electric Power University, Beijing 102206, People's Republic of China. [44] Peking University, Beijing 100871, People's Republic of China. [45] Qufu Normal University, Qufu 273165, People's Republic of China. [46] Shandong Normal University, Jinan 250014, People's Republic of China. [47] Shandong University, Jinan 250100, People's Republic of China. [48] Shanghai Jiao Tong University, Shanghai 200240, People's Republic of China. [49] Shanxi Normal University, Linfen 041004, People's Republic of China. [50] Shanxi University, Taiyuan 030006, People's Republic of China. [51] Sichuan University, Chengdu 610064, People's Republic of China. [52] Soochow University, Suzhou 215006, People's Republic of China. [53] South China Normal University, Guangzhou 510006, People's Republic of China. [54] Southeast University, Nanjing 211100, People's Republic of China. [55] State Key Laboratory of Particle Detection and Electronics, Beijing 100049, Hefei 230026, People's Republic of China. [56] Sun Yat-Sen University, Guangzhou 510275, People's Republic of China. [57] Suranaree University of Technology, University Avenue 111, Nakhon Ratchasima 30000, Thailand. [58] Tsinghua University, Beijing 100084, People's Republic of China. [59] Turkish Accelerator Center Particle Factory Group, (A)Istinye University, 34010, Istanbul, Turkey; (B)Near East University, Nicosia, North Cyprus, Mersin 10, Turkey. [60] University of Chinese Academy of Sciences, Beijing 100049, People's Republic of China. [61] University of Groningen, NL-9747 AA Groningen, The Netherlands. [62] University of Hawaii, Honolulu, Hawaii 96822, USA. [63] University of Jinan, Jinan 250022, People's Republic of China. [64] University of Manchester, Oxford Road, Manchester, M13 9PL, United Kingdom. [65] University of Muenster, Wilhelm-Klemm-Strasse 9, 48149 Muenster, Germany. [66] University of Oxford, Keble Road, Oxford OX13RH, United Kingdom. [67] University of Science and Technology Liaoning, Anshan 114051, People's Republic of China. [68] University of Science and Technology of China, Hefei 230026, People's Republic of China. [69] University of South China, Hengyang 421001, People's Republic of China. [70] University of the Punjab, Lahore-54590, Pakistan. [71] University of Turin and INFN, (A)University of Turin, I-10125, Turin, Italy; (B)University of Eastern Piedmont, I-15121, Alessandria, Italy; (C)INFN, I-10125, Turin, Italy. [72] Uppsala University, Box 516, SE-75120 Uppsala, Sweden. [73] Wuhan University, Wuhan 430072, People's Republic of China. [74] Xinyang Normal University, Xinyang 464000, People's Republic of China. [75] Yantai University, Yantai 264005, People's Republic of China. [76] Yunnan University, Kunming 650500, People's Republic of China. [77] Zhejiang University, Hangzhou 310027, People's Republic of China. [78] Zhengzhou University, Zhengzhou 450001, People's Republic of China. [a] Also at the Moscow Institute of Physics and Technology, Moscow 141700, Russia. [b] Also at the Novosibirsk State University, Novosibirsk, 630090, Russia. [c] Also at the NRC "Kurchatov Institute", PNPI, 188300, Gatchina, Russia. [d] Also at Goethe University Frankfurt, 60323 Frankfurt am Main, Germany. [e] Also at Key Laboratory for Particle Physics, Astrophysics and Cosmology, Ministry of Education; Shanghai Key Laboratory for Particle Physics and Cosmology; Institute of Nuclear and Particle Physics, Shanghai 200240, People's Republic of China. [f] Also at Key Laboratory of Nuclear Physics and Ion-beam Application (MOE) and Institute of Modern Physics, Fudan University, Shanghai 200443, People's Republic of China. [g] Also at State Key Laboratory of Nuclear Physics and Technology, Peking University, Beijing 100871, People's

Republic of China. [h] *Also at School of Physics and Electronics, Hunan University, Changsha 410082, China.* [i] *Also at Guangdong Provincial Key Laboratory of Nuclear Science, Institute of Quantum Matter, South China Normal University, Guangzhou 510006, China.* [j] *Also at Frontiers Science Center for Rare Isotopes, Lanzhou University, Lanzhou 730000, People's Republic of China.* [k] *Also at Lanzhou Center for Theoretical Physics, Lanzhou University, Lanzhou 730000, People's Republic of China.* [l] *Also at the Department of Mathematical Sciences, IBA, Karachi , Pakistan.*
